# Donor impurity related optical and electronic properties of cylindrical GaAs-Al_*x*_Ga_1−*x*_ As quantum dots under tilted electric and magnetic fields

**DOI:** 10.1038/s41598-020-65862-9

**Published:** 2020-06-08

**Authors:** Christian Heyn, C. A. Duque

**Affiliations:** 1 Center for Hybrid Nanostructures (CHyN), University of Hamburg, Luruper Chaussee 149, 22761 Hamburg, Germany; 2 Grupo de Materia Condensada-UdeA, Instituto de Física, Facultad de Ciencias Exactas y Naturales, Universidad de Antioquia UdeA, Calle 70 No. 52-21 Medellín, Colombia

**Keywords:** Materials science, Nanoscience and technology, Nanoscale devices, Nanoscale materials

## Abstract

This article makes a theoretical study of the optical and electronic properties in cylindrical GaAs-Al_*x*_ Ga_1−*x*_ As quantum dots in the presence of an arbitrarily located donor impurity and considering the simultaneous effects of tilted electric and magnetic fields. The studies are developed in the effective mass and parabolic band approximations. The solution of the Schrödinger equation is done through the finite element method considering tetrahedral meshes that can be adapted to regions where there are abrupt variations of the materials that make up the structure. Among the many results, reported for the first time in this article, we can mention: (*i*) the electronic spectrum, without and with shallow donor impurity, considering separate and combined effects of tilted electric and magnetic fields, (*ii*) the ground state binding energy as a function of the external electric and magnetic fields, their orientations concerning the axial axis of the quantum dot, and the impurity position, (*iii*) the squared reduced dipole matrix elements for impurity related inter-level optical transitions as a function of the tilted electric and magnetic fields and impurity position, and (*iv*) the optical absorption coefficient between the ground state and at least the first fifteen lowest excited states under tilted electric and magnetic fields and considering several impurity positions. From this study it can be concluded that the presence of tilted electric and magnetic fields and on-center or off-center shallow donor impurities, ostensibly enrich the optical and electronic properties of the system. It is observed that due to the rupture of the azimuthal symmetry of the cylindrical quantum dot, important modifications of the selection rules for inter-level transitions between states appear.

## Introduction

Theoretical and experimental reports about the study of charge carriers confined in III-V semiconductor quantum dots (QD) began to be known since the 1990s, including the most common GaAs/GaAlAs QDs^1–4^. In general, the reports consider the effective mass and parabolic band approximations^5–7^. A QD is a space region where, in general, the charge carriers are confined in two (2D-QDs) or three (3D-QDs) spatial directions. A 3D-QD is formed when a material with a certain energy gap is surrounded by another material with a larger energy gap^8,9^. The difference between the energy gaps, in type-I QDs, generates a potential well that confines the charge carriers (electrons and holes) within the QD region. Speaking particularly of 3D-QDs, due to the 3D-confinement, the solution of Schrödinger’s equation for a confined electron or hole leads to a discrete energy spectrum where the separation between energy levels increases as the size of the QD is reduced, that is, as the charge carriers confinement increases. The change of the CdTe QDs’ optical emission from red to green as the QD size decreases is well known^8,9^. One of the key factors in the study of 3D-QDs is the shape of the structure. In the last three decades, spherical, pyramidal, and core/shell QDs, among others, have been studied intensively^2,6,8–12^. For the confinement potentials, abrupt and parabolic models have been used to describe the spherical QDs, which with relative simplicity make it possible to obtain the electronic and hole energy spectrum^2,6^. The case of spherical QDs with parabolic confinement has an advantage that static electric and magnetic fields can be included in the problem with the possibility to find analytical solutions of the one-particle Scrhödinger equation^13–15^.

Another type of systems, to which researchers have paid considerable attention, are cylindrical quantum dots (CQD)^16–18^. These, depending on their height and radius, can be modeled as (*i*) 1D-systems, known as quantum wires, where the height of the cylinder is much greater than the radius of the structure, which is of the order of a few tens of the lattice parameter of the QD material and (*ii*) 2D-systems, known as quantum wells, where the height of the cylinder is comparable with a few tens of the lattice parameter of the cylinder material while its radius is of the order of several hundreds of the lattice parameter. Considering a CQD with an abrupt potential barrier between the dot material and the surrounding matrix, the Schrödinger equation solution presents serious problems accounting for the boundary conditions that arise from the connection between the circular and lateral faces of the cylinder. Writing the Schrödinger equation in cylindrical coordinates leads to a problem where the *ρ*- and *z*-dependent parts of the Hamiltonian are non-separable. To avoid this problem, the authors of multiple CQD-reports make use of infinite confinement potentials around the cylinder^10,19–21^. The confinement models with the combination of a finite confinement potential in one of the system’s directions (either radial or axial) and infinite potential in the other direction (axial or radial) have been proposed^22^. Finally, some widely used alternatives are the combination of two overlapping and independent parabolic potentials^23^ and a parabolic potential in one direction combined with an infinite barrier in the other one^24^.

Nedzinskas *et al*.^16^ have proposed an approximate model of variable separation to find the energy spectrum of an electron confined in a CQD with finite barrier confinement, taking into account the effective masses discontinuity. Their work, which can be easily extended to the study of quantum rods, has proven to be useful for the study of the ground and some excited states. Using the effective mass and parabolic band approximation, Sil *et al*.^17^ have reported a perturbative study of the binding energy of a donor impurity confined in an inhomogeneous CQD with a dielectric mismatch, finding that the binding energy of the ground state decreases with the CQD size and that the binding energy is maximum for on-axis impurities. A study of band structure, effective mass, band offset, and optical gain in GaInNAs/GaAs QD has been reported by Mal *et al*.^18^ using a 10-band $\overrightarrow{k}\cdot \overrightarrow{p}$ Hamiltonian, considering different concentrations of nitrogen and indium, and taking into account the effects of strain. Their main finding is that the optical gain increases with the decrease in the QD radius.

Once the electronic structure (that is the energies and their corresponding wave functions) for a confined charge carrier in a QD is known, the dipole matrix elements can be calculated. Together with the energies, they constitute the set of data necessary to study the linear and nonlinear optical properties associated with inter-level transitions. Some of the papers reported over the past two decades address issues such as: (*i*) the nonlinear optical rectification (NOR) and second harmonic generation (SHG) in QD with Kratzer-like potential^25^, (*ii*) the nonlinear optical absorption (NOA) in 4-level M-model QD^26^, (*iii*) the optical absorption (OA) in Morse-like QD under applied magnetic field and using a perturbative procedure^27^, (*iv*) the third order nonlinear optical susceptibility in InGaN/GaN QDs^28^, (*v*) the first and third order NOA and relative changes in the refractive index (RCRI) coefficients in CQD^29^, (*vi*) the impurity position dependent second order nonlinear optical susceptibility in CQD under external axial magnetic field and using a variational procedure^30^, (*vii*) the on-center impurity dependent NOA and RCRI coefficients in cylindrical nano-wire under combined effects of nonresonant intense laser field and axial stationary electric field and using the finite difference approximation^31^, (*viii*) the NOA coefficient in superlattices of spherical and cylindrical QDs immersed in a quantum wire and using the finite difference method^32^, (*ix*) the NOR in CQD under applied magnetic field, taking into account the Rashba spin-orbit interaction, and using a finite-difference discretization^33^, (*x*) the hydrostatic pressure effects on the diamagnetic susceptibility for a donor impurity confined in a CDQ under applied magnetic field^34^, and (*xi*) the intense laser field effects on the donor impurity binding energy in *δ*-doped CQD and using a variational technique^35^. In general, all of those works show that the asymmetries in the structures reinforce the dipole matrix elements and consequently the linear and nonlinear optical properties. Likewise, it is demonstrated that the presence of external fields constitutes an excellent tool through which red or blue shifts of the resonant peaks of the optical properties can be induced, which can be useful to modify, according to the need, the properties and response of optoelectronic devices. Researchers have also actively dabbled in linear and nonlinear optical properties that require a system of three and more electronic levels. An example is the well-known electromagnetic induced transparency (EIT) in which a physical system is subjected to the combined effect of two incident laser radiation. Detailed studies of the EIT can be found in refs. ^36–40^ where the effects of light polarization, hydrostatic pressure, temperature, applied magnetic field, parabolic confinement potentials, *V*- and Λ-type configurations for the involved energy levels, and spin-orbit interaction have been considered. Again, the presence of asymmetries in heterostructures is responsible for the magnification of EIT effect. It is important to note that, in the vast majority of the papers reported in the literature, including the ones cited here, the authors simplify the problem of confinement by making use of parabolic potentials for both the radial and axial direction of the CQD. With this approach, the authors avoid the problem of inseparable variables in the Scrhödinger equation. Additionally, this type of approach leads, in general, to analytical solutions for differential equations, even in the presence of electric and magnetic fields that preserve the axial symmetry of the structure.

The polaronic effects on CQD have also been reported in the literature. Using the Pekar type variational method under the condition of electric-LO-phonon and magnetic-LO -phonon strong coupling, Tiopsop *et al*.^41^ have developed a theoretical study of the time evolution of the polaron quantum mechanical state in asymmetric CQD qubit under an electromagnetic incident field. They treat the system in a QD as a two-level quantum qubit and study the influence of external EM-fields on the oscillation period of several electronâ€“LO-phonon coupling constants and different confinement lengths. In the same line of work, the effects of electric and magnetic fields as well as electron-polar optical phonon interaction on an electron bound to a Coulomb impurity in a CQD has been reported by Vartanian *et al*.^42^, finding that the corrections on the impurity binding energy due to the LO-phonons are significant and should be taken into account. The thermodynamic properties associated with electrons and impurities in CQD have also been studied in a significant number of recent articles. In particular, by using the Tsallis formalism, Khordad *et al*.^43,44^ have reported the entropy, the specific heat, and the internal energy in CQD with parabolic confinement potential under the effects of an axially applied magnetic field. Considering a hybrid parabolic and infinite confinement potential in a CQD, Gumber *et al*.^45^ calculated the thermal and magnetic properties of a CQD under externally applied electric and magnetic fields. The confinement model allowed the authors to obtain an analytical set of energies used to construct the partition function that made it possible to report the thermodynamic properties.

An exciton is a system that is obtained by introducing the Coulomb’s interaction between an electron and a hole. Depending on the spatial location of the two carriers, we can talk about spatially direct and indirect excitons^8,9^. In general, the 3D problem of an exciton confined in a QD implies a differential equation with six independent coordinates (three for the electron and three for the hole). Using appropriate changes in the coordinate system, it is possible to reduce the number of independent coordinates. Among the many variants that have been implemented is the combination of the Hylleraas coordinates combined with the variational method to obtain the ground state of a correlated electron-hole pair confined in a heterostructure^10^. El Hadi *et al*.^46^ have reported the externally applied electric field effects on the exciton binding energy in GaAs/GaAlAs CQD. They used a variational procedure with three parameters trial wave function; two parameters were used to take into account the geometrical confinement on the carriers whereas the third one was used to describe the electric field effects. Using Hylleraas coordinates combined with a variational procedure, within the effective mass and parabolic band approximations, the exciton related OA coefficient in cylindrical core/shell QD has been studied by El-Yadri and coauthors^47^.

Using the finite-difference method, the electric and magnetic field effects on the on-center and off-center impurity binding energy in CQD have been studied by Wang *et al*.^48^. In that work, the electric (magnetic) field has been considered perpendicular (parallel) to the axial axis of the structure. To reduce the complications of the problem, the authors used a parabolic confinement potential along the radial direction and implemented an approximated model of variable separation, which is a good approximation for structures where their dimensions are of the order of several effective Bohr radii of the QD material. Finally, the effects of tilted electric and magnetic fields on the ground state donor impurity binding energy in GaAs/AlGaAs CQD have been studied by Zeng *et al*.^49^. All their calculations were based on the potential morphing method within the framework of the effective mass approximation. They found that for parallel or perpendicular electric and magnetic fields, the binding energy magnetic shift is a monotonic function of the magnetic field strength.

A detailed review of the literature shows that to date there are no known theoretical reports (much less experimental) about the effects of tilted electric and magnetic fields on the electronic spectrum associated with a shallow donor impurity confined in a CQD. Consequently, the effects of such tilted fields on the on-center and off-center impurity related optical properties, such as OA coefficient, associated with intra-level transitions between confined states in the structure are also unknown. So, that is the spirit of this work. To study the effects of tilted electric and magnetic fields on the states of an electron confined in a CQD taking into account the effective mass changes between the QD and surrounding matrix regions. Next, a shallow donor impurity, arbitrarily located in the QD, is introduced; the full spectrum is recalculated and the ground state binding energy is obtained. Using the wave functions for the impurity problem, the squared dipole matrix elements between the ground state and at least fifteen excited states are calculated. Taking as input the squared dipole matrix elements and the energy spectrum, we obtain and discuss the OA coefficient for on-center and off-center impurities. All these studies have been made using the 3D-finite element method. It is important to note that nothing we discuss as a work topic in this article has been previously published in the literature. The organization of the paper is as follows: Section II contains the theoretical framework, in Section III, are discussed the obtained results for on-center and off-center impurities and, in Section IV, we report our conclusions.

## Theoretical framework

**Figure 1:**
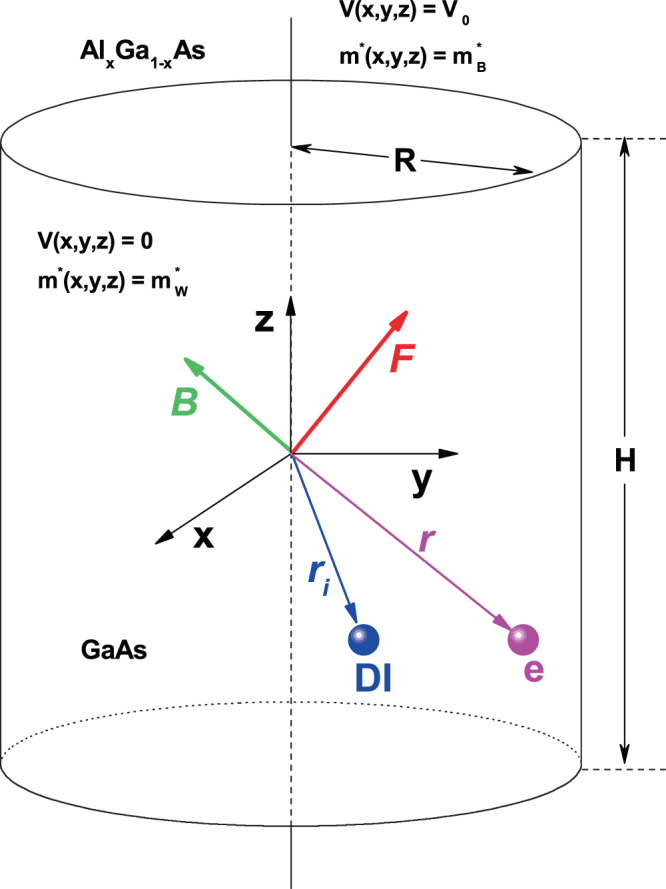
Pictorial view of the cylindrical GaAs-Al_*x*_ Ga_1−*x*_ GaAs quantum dot structure. The origin of coordinates is located at the gravity center of the cylinder. The dimensions of the structure are *R* for the radius and *H* for its height. The applied electric and magnetic fields, whose magnitudes are *F* and *B*, respectively, are indicated by the tilted arrows. The *V*(*x*, *y*, *z*)-confinement potential (m^*^(*x*, *y*, *z*)-electron effective mass) is zero (${m}_{W}^{\ast }$) inside the dot region and *V*_0_ (${m}_{B}^{\ast }$) elsewhere. The vectors ${\overrightarrow{r}}_{i}$ and $\overrightarrow{r}$ indicate the donor impurity (DI) and electron (e) positions.

Figure 1 shows the schematic view of the cylindrical GaAs-Al_*x*_ Ga_1−*x*_ GaAs quantum dot structure under applied electric and magnetic fields. The objective of this work is to study the energy states and their corresponding wave functions with the presence of on-center and off-center donor impurity atom in a cylindrical structure like the one shown in Fig. 1 and subjected to the effects of tilted stationary electric and/or magnetic fields.

Within the framework of the effective mass approximation, the Hamiltonian for this problem, in Cartesian coordinates, reads:

1 $$\begin{array}{rcl}H & = & \frac{1}{2{m}_{W,B}^{\ast }}{(i\hslash \overrightarrow{{\boldsymbol{\nabla }}}+e\overrightarrow{{\boldsymbol{A}}})}^{2}+e\overrightarrow{{\boldsymbol{F}}}\cdot \overrightarrow{{\boldsymbol{r}}}\\  &  & +\,V(x,y,z)-\frac{\eta {e}^{2}}{4\,\pi \,\varepsilon \,{\varepsilon }_{0}|{\overrightarrow{r}}_{i}-\overrightarrow{r}|},\end{array}$$

where *e* is the electron charge, ${m}_{W,B}^{\ast }$ is the effective mass (***B*** means the Al_*x*_ Ga_1−*x*_ GaAs barrier region and ***W*** corresponds to the GaAs quantum dot material), *V*(*x*, *y*, *z*) is the confinement potential which is zero inside the dot region and *V*_0_ elsewhere, *ε* is the GaAs static dielectric constant, *ε*_0_ is the vacuum permittivity, and |*r*_*i*_ − *r*| is the electron-impurity distance (the impurity and electron positions are given by ${\overrightarrow{r}}_{i}=({x}_{i},{y}_{i},{z}_{i})$ and $\overrightarrow{r}=(x,y,z)$, respectively). Here, η = 0 when there is not the impurity center and η = 1 when the impurity has been considered.

The gauge chosen to describe the magnetic field effect implies that the magnetic vector potential must satisfy the following two conditions: (*i*) $\overrightarrow{{\boldsymbol{\nabla }}}\cdot \overrightarrow{{\boldsymbol{A}}}=0$ and (*ii*) $\overrightarrow{{\boldsymbol{A}}}=-\,\frac{1}{2}\overrightarrow{{\boldsymbol{r}}}\times \overrightarrow{{\boldsymbol{B}}}$. Under the condition (*i*), the Hamiltonian in Eq. (1) takes the form:

2 $$\begin{array}{rcl}H & = & -\frac{{\hslash }^{2}}{2{m}_{W,B}^{\ast }}{\overrightarrow{{\boldsymbol{\nabla }}}}^{2}+\frac{ie\hslash }{{m}_{W,B}^{\ast }}\overrightarrow{{\boldsymbol{A}}}\cdot \overrightarrow{\nabla }+\frac{{e}^{2}}{2{m}_{W,B}^{\ast }}{\overrightarrow{{\boldsymbol{A}}}}^{2}\\  &  & +\,e\overrightarrow{{\boldsymbol{F}}}\cdot \overrightarrow{{\boldsymbol{r}}}+V(x,y,z)-\frac{\eta {e}^{2}}{4\,\pi \,\varepsilon \,{\varepsilon }_{0}|{\overrightarrow{r}}_{i}-\overrightarrow{r}|}.\end{array}$$

In this work, the electric and magnetic fields will be considered in the *y* = 0 plane. It means, $\overrightarrow{{\boldsymbol{B}}}=({B}_{x},{B}_{y},{B}_{z})=B(\cos \,\theta ,0,\,\sin \,\theta )$ and $\overrightarrow{{\boldsymbol{F}}}=({F}_{x},{F}_{y},{F}_{z})=F(\cos \,\alpha ,0,\,\sin \,\alpha )$, where *θ* and *α* are the angles of $\overrightarrow{{\boldsymbol{B}}}$ and $\overrightarrow{{\boldsymbol{F}}}$ with respect to the *x*-axis, respectively. Considering the previous condition (*ii*), the Eq. (2) is transformed into the following expression

3 $$\begin{array}{rcl}H & = & -\frac{{\hslash }^{2}}{2{m}_{W,B}^{\ast }}{\overrightarrow{{\boldsymbol{\nabla }}}}^{2}-\frac{i\,e\hslash }{{m}_{W,B}^{\ast }}\left[y{B}_{z}\frac{\partial }{\partial x}+(z{B}_{x}-x{B}_{z})\frac{\partial }{\partial y}-y{B}_{x}\frac{\partial }{\partial z}\right]\\  &  & +\,\frac{{e}^{2}}{8{m}_{W,B}^{\ast }}[{x}^{2}\,{B}_{z}^{2}+{y}^{2}\,({B}_{x}^{2}+{B}_{z}^{2})+{z}^{2}\,{B}_{x}^{2}-2\,x\,z\,{B}_{x}\,{B}_{z}]\\  &  & +\,e\,(x\,{F}_{x}+z\,{F}_{z})+V(x,y,z)-\frac{\eta \,{e}^{2}}{4\,\pi \,\varepsilon \,{\varepsilon }_{0}|{\overrightarrow{r}}_{i}-\overrightarrow{r}|}.\end{array}$$

The energies and wave functions of the bound states can be obtained by solving the Schrödinger equation:

4 $$H{\Psi }_{i}(x,y,z)={E}_{i}{\Psi }_{i}(x,y,z).$$

The energies and wave functions corresponding to the Hamiltonian in Eq. (3) are calculated with the *COMSOL-Multiphysics*^50^ software, which uses a FEM to solve numerically the partial differential equation. A complete description of the *COMSOL-Multiphysics* licensed software that includes the foundation of the FEM, the construction of meshes, the discretization of the differential equations, the methods to optimize the processes, the construction of geometries, and the convergence criteria can be found in^51,52^. Since Ψ_*i*_(*x*, *y*, *z*) is finite, the Dirichlet boundary condition implies that any of its values far away are equal to zero. For layered structures such as the one in the current study, the Schrödinger equation interface accounts for the discontinuity in the effective mass by implementing the BenDaniel-Duke boundary conditions.

The optical absorption coefficient (OAC) to be evaluated in this work comes from the imaginary part of the dielectric susceptibility, and is given by^53–55^:

5 $$\alpha ({E}_{P})=\left(\frac{\pi \,\sigma \,{E}_{P}}{\hslash \,c\,\sqrt{\varepsilon }\,{\varepsilon }_{0}}\right)\,\mathop{\sum }\limits_{f=1}^{15}{|{\mu }_{\overrightarrow{\xi }}^{f,0}|}^{2}\,\delta ({E}_{f0}-{E}_{P}),$$

where *E*_*P*_ is the incident radiation energy, *σ* = 3 × 10^23^ cm^−3^ is the electron density, and the quantity *E*_*f*0_ = *E*_*f*_ − *E*_0_ is the energy difference between the initial (*E*_0_) and final (*E*_*f*_) states of the light-induced inter-level transition. In order to take into account all the possible damping effects associated with inter-level transitions induced by photon absorption, the Dirac delta function is usually substituted by a Lorentzian one via the following expression

6 $$\delta \,({E}_{f0}-{E}_{P})\cong \frac{1}{\pi }\,\frac{\Gamma }{{({E}_{f0}-{E}_{P})}^{2}+{\Gamma }^{2}},$$

where Γ (=5 meV in this work) accounts for the energy associated to the corresponding damping rates. In this work we deal with the low temperature regime (*T* = 4 K) and consequently, in the OAC process, when there is not incident radiation only the ground state is occupied. Finally, $\overrightarrow{\xi }$ is the unit vector representing the polarization of the —homogeneously intense— incident light. For right (left) hand circularly polarized light in the *xy*-plane, $\overrightarrow{\xi }=({\overrightarrow{e}}_{1}-i{\overrightarrow{e}}_{2})/\sqrt{2}$ [$\overrightarrow{\xi }=({\overrightarrow{e}}_{1}+i{\overrightarrow{e}}_{2})/\sqrt{2}$] and for *z*-polarized light, $\overrightarrow{\xi }={\overrightarrow{e}}_{3}$. Here, ${\overrightarrow{e}}_{1}$, ${\overrightarrow{e}}_{2}$, and ${\overrightarrow{e}}_{3}$ are the unit vectors along the *x*-, *y*-, and *z*-directions, respectively.

The general expression for the electric dipole moment matrix element, ${\mu }_{\overrightarrow{\xi }}^{f,i}$, is the following:

7 $${\mu }_{\overrightarrow{\xi }}^{f,i}=\langle {\Psi }_{f}|e\,\overrightarrow{\xi }\cdot \overrightarrow{r}|{\Psi }_{i}\rangle ,$$

where $\overrightarrow{r}$ is the vector position. From here on we will use the following notation for the reduced dipole matrix elements: ${P}_{+,-}^{f,i}=\frac{1}{e}\,{\mu }_{({\overrightarrow{e}}_{1}\mp i\,{\overrightarrow{e}}_{2})/\sqrt{2}}^{f,i}$ and ${P}_{z}^{f,i}=\frac{1}{e}\,{\mu }_{{\overrightarrow{e}}_{3}}^{f,i}$

The incident photon energy corresponding to the resonant peak of each energy transition is given by ${E}_{P}^{R}=\sqrt{{E}_{f0}^{2}+{\Gamma }^{2}}$. Under the condition ${E}_{f0}^{2}\gg {\Gamma }^{2}$ the magnitude of the OAC at the resonant peak is

8 $${\alpha }^{R}=\frac{\sigma }{\hslash \,c\,\sqrt{\varepsilon }\,{\varepsilon }_{0}}{|{\mu }_{\overrightarrow{\xi }}^{f,0}|}^{2}\,\left(\frac{{E}_{f0}}{\Gamma }+\frac{1}{4}\right).$$

In those cases where ${E}_{f0}\gg \Gamma /4$, the magnitude of the resonant peak essentially depends on the product ${E}_{f0}{|{\mu }_{\overrightarrow{\xi }}^{f,0}|}^{2}$.

Next, in Sec. III we proceed to present our results with their corresponding discussions. In Sec. III.A. we discuss the particular case of on-center impurities whereas in Sec. III.B. we analyze the most general case corresponding to off-center impurity effects.

## Results and Discussion

### On-center donor impurity

**Figure 2:**
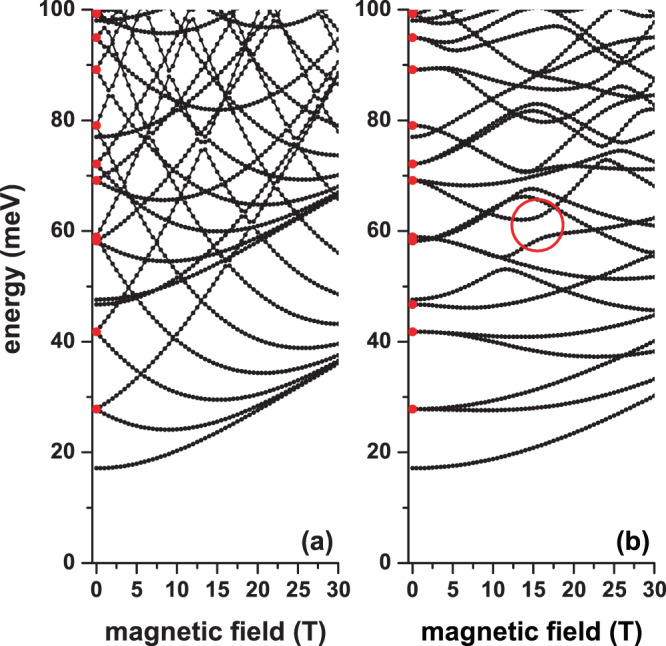
Energy spectra for a confined electron in a cylindrical GaAs-Al_0.3_ Ga_0.7_ As quantum dot as a function of the applied magnetic field with *θ* = *π*/2 (**a**) and *θ* = 0 (**b**). The results are for *η* = 0, *F* = 0, *R* = 20 nm, and *H* = 20 nm. The solid red circles at *B* = 0 identify double degenerate states at zero magnetic fields. The open red circle in (**b**) identifies the anti-crossings between two states.

In Fig. 2, we present the energies of some of the lowest states for an electron confined to a CQD as a function of the applied magnetic field. The results are for zero electric fields without impurity effects. In (a) the magnetic field is applied in the *z*-direction, which allows preserving the axial symmetry of the structure. In (b), where the magnetic field is applied in the *x*-direction, the axial symmetry is clearly destroyed. The reason why at zero magnetic fields, in both figures, some states doubly degenerate comes from the QD cylindrical symmetry. For example, at zero magnetic fields, the first and second excited states are degenerate and they are associated with *p*_*x*_- and *p*_*y*_-like states, that is, their wave functions are odd functions along the *x*/*y* axis for the first/second excited state. In (a), the fifth and sixth excited states are not degenerate states at zero magnetic fields. The fifth state corresponds to an *s*-like one in the *xy*-plane whereas the sixth excited state clearly shows a *p*_*z*_-like symmetry coming from the *z*-confinement. In the presence of an applied magnetic field, in (a) each pair of degenerate states separates into an ever-increasing energy state (which is associated with the values *l* = +1, +2, +3, *â*, where *l* is the magnetic quantum number) and in another state that initially decreases and then grows asymptotically to approach the state with *l* = 0 (these mixed behavior states are associated with the values *l* = −1, −2, 3…). It is observed that regardless of the applied magnetic field, both in (a) and in (b), the ground state does not show crossings with other states, which indicates that there are no oscillations of the ground state symmetry. The crossings between different curves, both in (a) and in (b), are associated with accidental degenerations. This kind of degeneration comes from the proper combination of the dimensions of the structure and the intensity of the applied magnetic field and they have nothing to do with the Hamiltonian symmetries of the problem. Proper of the magnetic field along the *x*-axis, the presence of anti-crossings between some states can be seen in (b), such as the one highlighted by the red circle at *B* = 15 T and *E* = 62 meV. In these  anti-crossings, one state that, depending on the magnetic field, shows an energy decreasing behavior, exchanges its symmetry and its decreasing character with one state that is a growing energy function with the magnetic field. Comparing (a) with (b), it is clearly observed that in the first case, the states are more sensitive to the magnetic field with respect to what is presented in the second case. Here it is worth remembering that our CQD has a radius that is equal to its height. This, in reality, implies that the confinement along the *z*-direction is much greater with respect to what is experienced on the *xy*-plane. The quasi-linear behavior of the ground state in (a) for *B* =30 T implies that for this particular value, the parabolic confinement associated with the magnetic field dominates over the QD potential. In (b), it is noted that even at *B* = 30T, the ground state fails to reach this linear character, which is interpreted with the fact that for such field intensity value, even the potential at the barriers in *z* = ±*H*/2, competes with the magnetic field parabolic potential.

**Figure 3:**
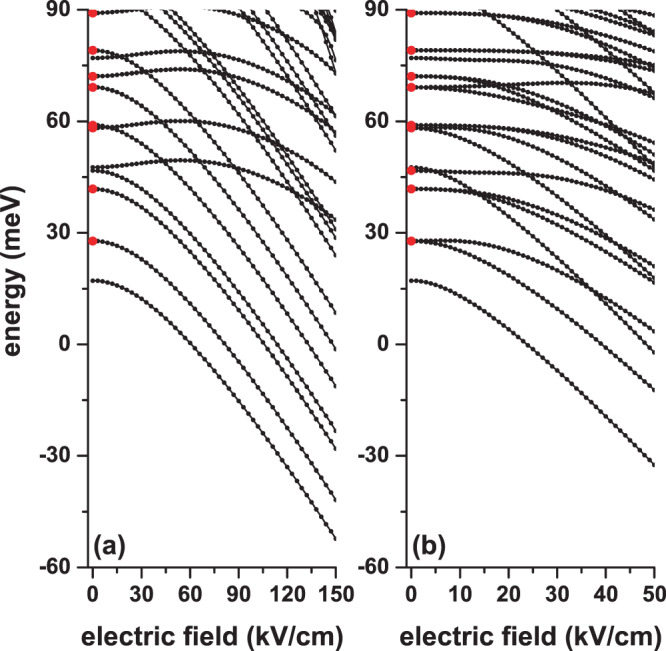
Energy spectra for a confined electron in a cylindrical GaAs-Al_0.3_Ga_0.7_ As quantum dot as a function of the applied electric field with *α* = *π*/2 (**a**) and *α* = 0 (**b**). The results are for *η* = 0, *B* = 0, *R* = 20 nm, and *H* = 20 nm. The solid red circles at *F* = 0 identify double degenerate states at zero electric field.

In Fig. 3, we present the energies of the lowest states for an electron confined to a CQD as a function of the applied electric field. The results are for zero magnetic fields without impurity effects. In (a) the electric field is applied in the *z*-direction, which allows preserving the axial symmetry of the structure. In (b), where the electric field is applied in the *x*-direction, the axial symmetry is clearly destroyed but the reflection one with respect to the z = 0 plane is preserved. In both figures, at *F* = 0, the states present the same degenerations as those reported in Fig. 2. In Fig. 3(a), with electric field along the *z*-direction, note that, despite the presence of the electric field, the states preserve their degeneration. This is because all the degenerate states in that figure correspond to even or odd wave functions with respect to the *x* = 0 or *y =* 0 planes, a situation that is preserved even in the presence of the electric field. When applying the electric field along the *x*-direction, Fig. 3(b), it is observed that all degenerations are broken since the odd or even symmetry of the wave functions for reflections in the *x* = 0 plane is destroyed. While in Fig. 3(a) the electric field acts along a region whose length is essentially 20 nm, which corresponds to the cylinder height, in Fig. 3(b) the action of the field is over a distance of 40 nm, corresponding to the diameter of the structure. This explains the reason why while a 120 kV/cm field in Fig. 3(a) manages to induce a Stark shift of −45 meV on the ground state, the same effect is observed in Fig. 3(b) with a much lower electric field of only 50 kV/cm. The decreasing behavior of most states is associated with the shift towards lower energies of the bottom of the potential well in the presence of the applied electric field. For sufficiently high electric fields, all states acquire a decreasing linear behavior due to the combined effect of the reduction in the effective width of the triangular well and the shift towards lower energies of its minimum energy. Figure 3 shows some states that present a mixed behavior as a function of the applied electric field: first they increase with energy, next they reach a maximum and finally they decrease; an example of this behavior is shown by the sixth excited state in Fig. 3(a), with Ψ_7_ wave function. An analysis of Ψ_7_ shows that, for that particular state, when *F* = 0, the wave function is odd with respect to the *z* = 0 plane, which is equivalent to say that Ψ_7_ has two antinodes along the *z*-axis. When the electric field is turned on, for example at *F = 30* kV/cm, the two nodes of Ψ_7_ move along the *z*-axis in the opposite direction to the field. This is interpreted as a greater location of the state and manifests as an increase in energy. For *F* = 60 kV/cm a change of symmetry appears on Ψ_7_, in which case the wave function has only one antinode along the *z*-axis and a multiple set of antinodes on the *xy*-plane. For larger electric fields, the previous symmetry is preserved, but the location of Ψ_7_ towards the *z* = −*H*/2 barrier is increased, resulting in an increase of the energy that is overlapped by the effect of displacement towards low energies of the potential barrier bottom. In that case, Ψ_7_ acquires the same decreasing behavior in energy with the applied electric field, as shown for example by the ground and first two states excited in Fig. 3(a). It is important to say that the analysis shown here can be extended to Fig. 3(b) even though the field is applied perpendicularly to the axial axis of the cylinder.

**Figure 4:**
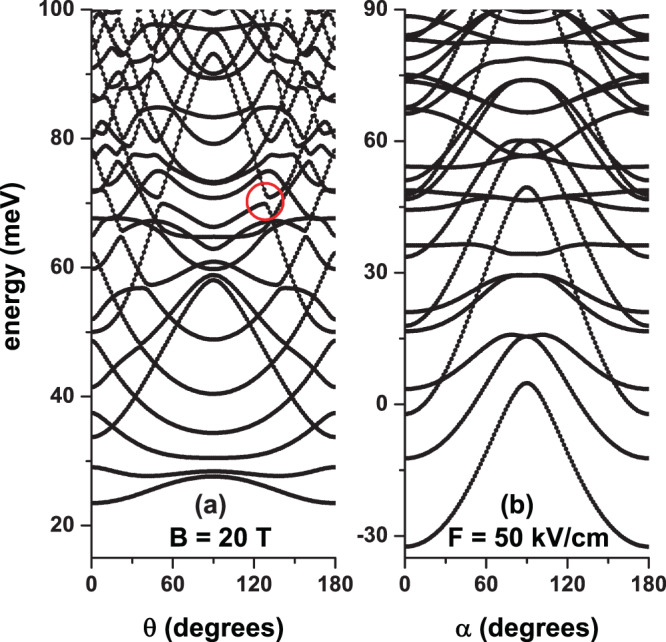
Energy spectra for a confined electron in a cylindrical GaAs-Al_0.3_ Ga_0.7_ As quantum dot as a function of the *θ*-angle with *B* = 20 T and *F* = 0 (**a**) and as a function of the *θ*-angle with *F* = 50 kV/cm and *B* = 0 (**b**). The results are for *η* = 0, *R* = 20 nm, and *H* = 20 nm. The open red circle in (**a**) identifies the  anti-crossings between two states.

In Fig. 4, we present the energies of some of the lowest states for an electron confined in a CQD as a function of the *θ*-angle with *B* = 20 T and *F* = 0 (a) and as a function of the *α*-angle with *F* = 50 kV/cm and *B* = 0 (b). When comparing the two panels of the figure, it is observed that for *θ* = *α* = *π*/2 in Fig. 4(a), there is a complete absence of degenerations coming from Hamiltonian symmetries while in Fig. 4(b) a set of doubly degenerated states appears. In that particular case, the axial magnetic field (*B* = 20 T, with *θ* = *π*/2) unfolds the states with *l* ≠ 0. For that kind of states (with *l* = ±1, ±2, ±3, …) the axial electric field (*F* = 50 kV/cm, with *α* = *π*/2) simply implies that the wave functions shift towards the *z* = −*H*/2 region, without changing their symmetries. Additionally, in both panels, a complete degeneration rupture is observed for *θ* = *α* = 0 and *θ* = *α* = *π*. In this case, both fields are applied perpendicularly to the axial axis of the cylinder giving rise to breakage of all symmetries in the Hamiltonian. In both panels, the crossings between energy curves are identified with accidental degenerations that cannot be associated with any kind of Hamiltonian symmetry. In Fig. 4(a), the  anti-crossings between states can be seen, one is highlighted with a red circle. Again, this type of  anti-crossings, which means an exchange of symmetry in the wave functions of these states, does not come from a problem symmetry since the solution of the differential equation for a specific value of *θ* is completely independent of the solution obtained for a greater or lesser value of the angle. In other words, what we are saying is that the spectrum obtained for a certain angle value is completely independent of the energy spectrum of a different angle value. In Fig. 4(b), the absence of  anti-crossings between states is noted.

**Figure 5:**
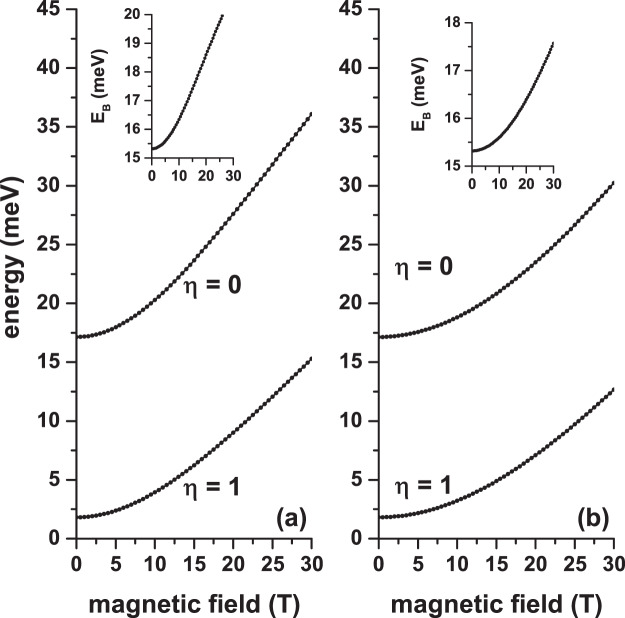
Ground state energy in a cylindrical GaAs-Al_0.3_ Ga_0.7_ As quantum dot as a function of the applied magnetic field with *θ* = *π*/2 (**a**) and *θ* = 0 (**b**). The results are with (*η* = 1) and without (*η* = 0) shallow donor impurity for *F* = 0, *R* = 20 nm, and *H* = 20 nm. The insets show the corresponding ground state binding energy.

**Figure 6:**
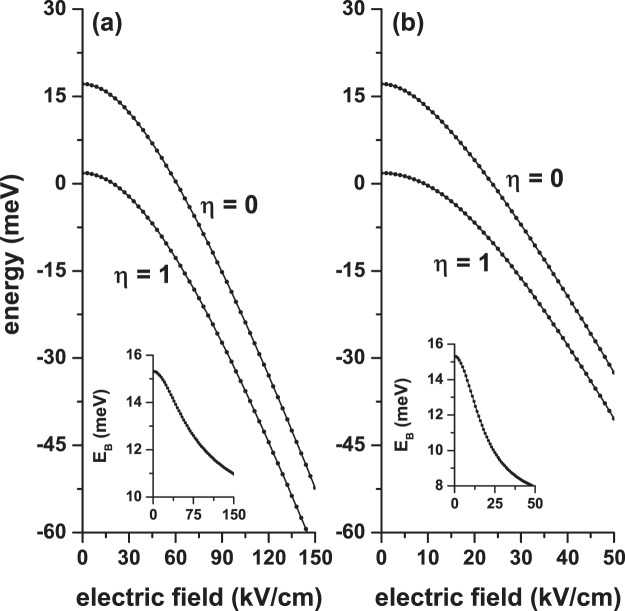
Ground state energy in a cylindrical GaAs-Al_0.3_ Ga_0.7_ As quantum dot as a function of the applied electric field with *α* = *π*/2 (**a**) and *α* = 0 (**b**). The results are with (*η* = 1) and without (*η* = 0) shallow donor impurity for *B* = 20, *R* = 20 nm, and *H* = 20 nm. The insets show the corresponding ground state binding energy.

**Figure 7:**
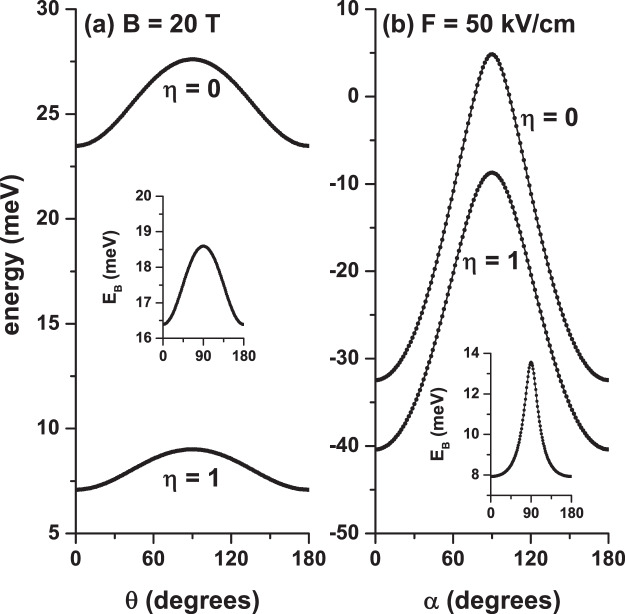
Ground state energy in a cylindrical GaAs-Al_0.3_ Ga_0.7_ As quantum dot as a function of the *θ*-angle with *B* = 20 T and *F* = 0 (**a**) and as a function of the *α*-angle with *F* = 50 kV/cm and *B* = 0 (**b**). The results are with (*η* = 1) and without (*η* = 0) shallow donor impurity for *R* = 20 nm and *H* = 20 nm. The insets show the corresponding ground state binding energy.

Once the energy spectrum is obtained for an electron confined in a GaAs-Al _0.3_ Ga_0.7_ As CQD under either separate or combined electric and magnetic fields effects, we proceed to include in the problem the presence of an on-center donor impurity. In Figs. 5, 6, and 7 we present our results for the ground state energy as a function of the applied magnetic and electric fields and the angle that these fields have concerning the *x*-axis, respectively. The results are presented with the same configurations that were chosen in Figs. 2, 3, and 4. In the inset of each figures the calculated ground state binding energy is included. The impurity binding energy is obtained from the difference between the ground state without impurity and the same state in the presence of impurity. For example, the inset of Fig. 5(a) is obtained from the difference between the ground states of Figs. 2 and 5.

In Figs. 5, 6, and 7 it is seen that in the presence of impurity (*η* = 1) the ground state presents a shift towards lower energies (red shift). This is in line with the negative sign of the last term in Eq. (3). Of course, this shift is not rigid, as shown by the non-constant behavior of the binding energy in each of the insets. However, one can see that with *η* = 1 the ground state follows the same behavior that occurs for *η* = 0: increasing in the presence of the magnetic field (Fig. (5)), decreasing in the presence of the electric field (Fig. (6)), and mixed behavior — increasing for angles between zero and *π*/2 and decreasing for angles between *π*/2 and *π* (Fig. (7)). In both panels of Fig. 5 the binding energy grows because the magnetic field imposes an additional confinement on the system, which can be seen as a reduction in the effective size of the QD dimensions. In that case, the electronic wave function is pushed towards the QD center, where the impurity has been located, producing an effective decrease in the electron-impurity distance, thereby reinforcing the Coulomb interaction. The two panels in Fig. 6 show that the binding energy decreases with the electric field. In Fig. 6(a) the electric field induces the creation of a dipole, oriented along the *z*-axis, where for very high electric field strengths the maximum distance between the two charges is *d*_*max*_ = *H*/2 = 10 nm. In the case of Fig. 6(b), we obtain *d*_*max*_ = *R* = 20 nm. This fact explains the reason why the two insets of Fig. 6 demonstrate that the rate of binding energy decrease diminishes for high values of the electric field and justify, additionally, that in Fig. 6(b) with only *F* = 50 kV/cm the Stark shift of the ground state is approximately double of that obtained in Fig. 6(a) with *F* = 150 kV/cm.

The results are shown in Fig. 7 are in full agreement with the ones of Figs. 5 and 6, discussed above. The binding energy is maximum when the two fields, which in this case are of constant magnitude, are applied in the *z*-direction. The key question in Fig. 7(a) is, why does the energy increase as the *θ*-angle ranges from 0 to *π*/2? Below we present how this situation can be interpreted. The magnetic field, of constant magnitude, in this case, generates a cylindrical region that confines the carriers and which have essentially a constant radius. The axial axis of such cylindrical region is located around an axis parallel to the field. As *θ* goes from zero to *π*/2, the maximum height of that cylinder changes from *h*_*max*_ = 2*R* = 40 nm to *h*_*max*_ = *H* = 20 nm. This can be seen as a decrease in the volume of the region where the electron is confined, thus translating into an increase in binding energy. In the case of Fig. 7(b), the increase in binding energy as *α* goes from zero to *π*/2 is since in this range of variations of the angle there is a decrease of the region where the field acts — the length of this region goes from *r*_*max*_ = 2*R* = 40 nm to *r*_*max*_ = *H* = 20 nm whereby the effects of the field are attenuated. In other words, as the field passes from *α* = 0 to *α* = *π*/2, the dipole moment of the two charges decreases producing a reinforcement of the Coulomb interaction and eventually leading to an increase in binding energy.

**Figure 8:**
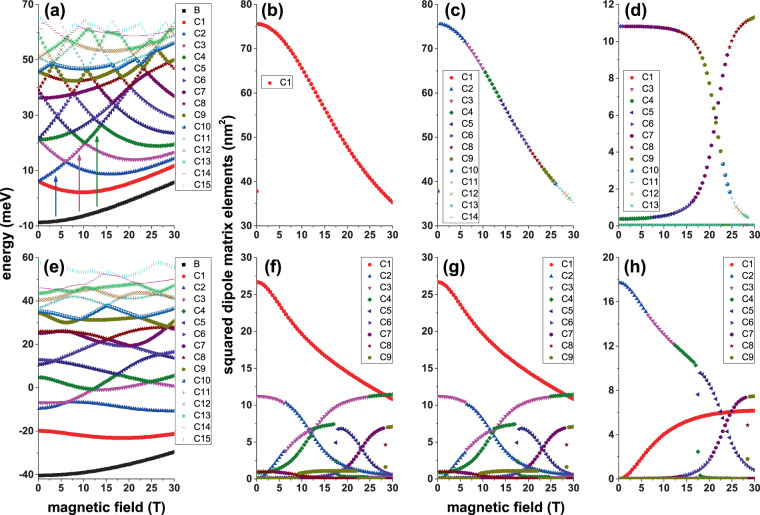
Energy of the on-center impurity lowest confined states (**a**,**e**) and Ψ_0_ → Ψ_*n*_ (*n* = 1, 2, 3, …) squared reduced dipole matrix elements (**b**–**d**, **f**–**h**) in a cylindrical GaAs-Al_0.3_ Ga_0.7_ As quantum dot as a function of the applied magnetic field with *θ* = *α* = *π*/2 (**a**–**d**) and *θ* = *α* = 0 (**e**–**h**). The results are for *F* = 50 kV/cm, *R* = 20 nm, and *H* = 20 nm. In (**a**,**e**) the label *B* identifies the ground state whereas the labels *Cn* (*n* = 1, 2, 3, …) are for the excited states. In the squared reduced dipole matrix elements figures, the labels *Cn* (*n* = 1, 2, 3, …) identify the Ψ_0_ → Ψ_*n*_ transitions. The polarizations of the incident photon are as follows: left hand circular polarized radiation-|*P*_−_|^2^ (**b**,**f**), right hand circular polarized radiation-|*P*_+_|^2^ (**c**,**g**), and *z*-linear polarized radiation-|*P*_*z*_|^2^ (**d**,**h**).

After the energy spectrum and wave functions have been obtained for an electron confined in a GaAs-Al_0.3_ Ga_0.7_ As CQD in the presence of an on-center impurity, we have the necessary input data to calculate, for example, the optical properties. There are many variants of interest; from calculating the absorption and relative changes in the refraction index coefficients —considering the first and third-order terms— through nonlinear optical rectification, and ending in the second and third harmonic generation coefficients. It is also possible to calculate the electromagnetic induced transparency and the impurity related Raman scattering. All these optical properties require two basic elements: (*i*) the energy spectra and (*ii*) the squared dipole matrix elements for different polarizations of the incident and secondary radiation (the latter in the case of the electron or impurity related Raman scattering) that are calculated using Eq. (7). In this case, we only require as input information the wave functions of the involved states. Clearly, the behavior and values of the squared dipole matrix elements depend on the polarization of the incident or scattered radiation and the wave functions symmetries. In Fig. 8, in panels 8(a) and 8(e), we show the dependence on the applied magnetic field of the energy spectrum for an electron confined in a CQD with an on-center impurity considering a constant electric field (*F* = 50 kV/cm). In Fig. 8(a) the two fields are applied in the *z*-direction while in Fig. 8(b) both fields are directed in the *x*-direction. The other panels in the figure show the results for squared reduced dipole matrix elements as a function of the applied magnetic field considering incident radiation with right-hand circular polarization 8(b,f), left-hand circular polarization 8(c,g), and *z*-directed linear polarization 8(d,h). The presence of the electric field in Fig. 8(a) guarantees the breaking of the reflection symmetry concerning the *z* = 0 plane. In Fig. 8(b) both fields, applied each separately, lead to breaking the symmetry of the system. When analyzing the squared reduced dipole matrix elements $({|{M}_{\overrightarrow{\xi }}^{f,i}|}^{2}={|{\mu }_{\overrightarrow{\xi }}^{f,i}/e|}^{2})$ it should be taken into account that except for *B* = 0 and the ground state, in general, the Hamiltonian eigenfunctions simultaneously have real and imaginary components, even in the case of *F* = 0. We emphasize that in the presence of an electric field, the lifetime of the states is infinite because the Dirichlet boundary conditions are used for a large cylinder whose radius is 30 nm and height 40 nm, that is a sufficiently large and concentric cylinder with the CQD of interest being positioned in the center. Understanding the reduced dipole matrix elements implies knowing previously the symmetries of the real and imaginary components of the wave functions. Let us take as an example the results shown in Fig. 8(b), which corresponds to *θ* = *α* = *π*/2. The analysis of the wave functions shows that: (*i*) Ψ_0_ is always a real function and is simultaneously an even function with respect to the *x* = 0 and *y* = 0 planes; additionally, it presents an antinode along the *z*-direction that is displaced towards the *z* = −*H*/2 region, (*ii*) the wave function Ψ_1_ has real and imaginary components that are not null; particularly $\Re ({\Psi }_{1})$ [$\Im ({\varPsi }_{1})$] is an odd/even function for reflections in the *x* = 0/*y* = 0 [*y* = 0/*x* = 0] planes. Both components of Ψ_1_ only present an antinode along the *z*-direction which, like the ground state, is also shifted to *z* = −*H*/2, *iii*) the effect of the magnetic field on Ψ_0_ and Ψ_1_ only manifests in the location of these two states that moves towards the axial axis region of the QD. This results in an increase in the overlapping of wave functions. Simultaneously, the overlap between the product of the wave functions and the *x* or *y* linear functions that appear in the reduced dipole matrix elements decreases. This explains the behavior shown by the results in Fig. 8(b). Using the symmetry conditions of the wave functions, in Fig. 8(b) it is true that ${|{M}_{({\overrightarrow{e}}_{1}-i{\overrightarrow{e}}_{2})/\sqrt{2}}^{\mathrm{1,0}}|}^{2}={(\langle {\varPsi }_{0}|x|\Re ({\varPsi }_{1})\rangle +\langle {\varPsi }_{0}|y|\Im ({\varPsi }_{1})\rangle )}^{2}$. The parity of Ψ_0_ and Ψ_1_ with respect to the *x* = 0 and *y* = 0 planes is responsible for the null value of ${|{M}_{{\overrightarrow{e}}_{3}}^{1,0}|}^{2}$ in Fig. 8(d). Similar analyzes can support the other results of ${|{M}_{\overrightarrow{\xi }}^{f,i}|}^{2}$. Comparison between Fig. 8(a,b) may lead to the conclusion that the incident radiation with right or left circular polarization excites states with very different energies but with identical dipole matrix elements. This will be reflected in totally different behaviors of the absorption peaks. Figure 8(f,g) allow us to conclude that, regardless of the direction of the circular polarization of the incident radiation, when the electric and magnetic fields are perpendicular to the axial axis of the CQD, the dipole matrix elements are the same. Finally, comparing the two rows of Fig. 8, it is clear that the symmetry break implies an enrichment of the structures that will be observed in the absorption spectra.

**Figure 9:**
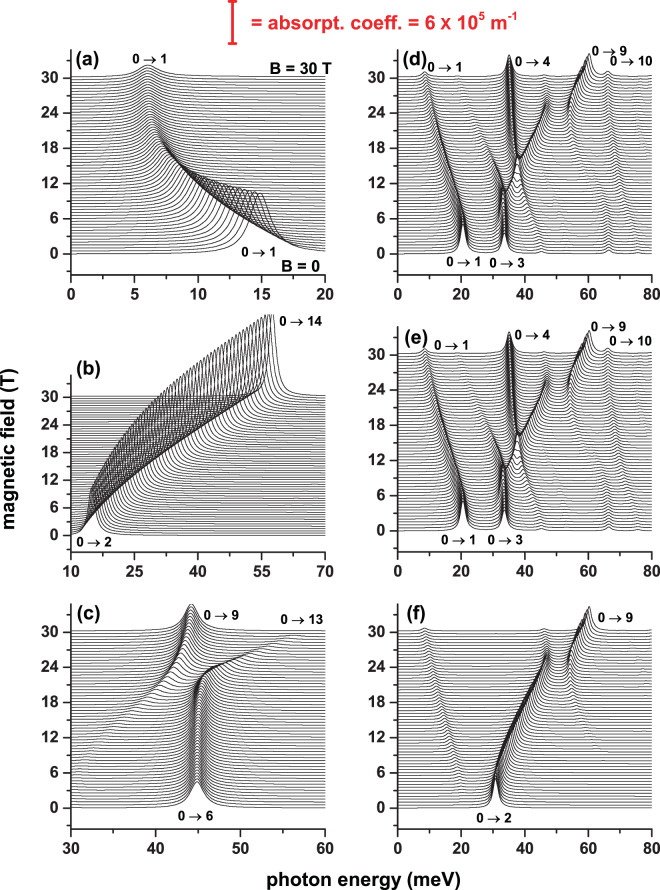
On-center donor impurity related optical absorption coefficient in a cylindrical GaAs-Al_0.3_ Ga_0.7_ As quantum dot as a function of the incident photon energy, and the applied magnetic field, with *θ* = *α* = *π*/2 (**a**–**c**) and *θ* = *α* = 0 (**d**–**f**). The results are for *F* = 50 kV/cm, *R* = 20 nm, and *H* = 20 nm. The vertical red bar at the top indicates the magnitude of the optical absorption coefficient. The polarizations of the incident photon are as follows: left hand circular polarized radiation (**a**,**d**), right hand circular polarized radiation (**b**,**e**), and *z*-linear polarized radiation (**c**,**f**). In all panels, the symbols 0 → *n*, with *n* = 1, 2, 3, … (where 0 corresponds to the initial ground state and *n* is for the final excited one), indicate the allowed optical transitions at *B* = 0 (at the bottom of each panel) and *B* = 30 T (at the top of each panel).

As an example of the possible applications that can be implemented with the information obtained for the energy spectra and the squared dipole matrix elements, in Fig. 9 we report the impurity related OAC in GaAs-Al_0.3_ Ga_0.7_ As QD as a function of the incident photon energy considering a fixed value of an applied electric field for magnetic field strengths between zero and 30T. Both fields have been chosen parallel and perpendicular to the axial direction. All the information necessary to obtain the OACs appears in Fig. 8, that is: the energies of the initial state (ground state) and final states and the squared reduced dipole matrix elements, all of them for the transitions from the ground state to at least the first fifteen excited states. In Fig. 8(b) it is noted that only ${|{P}_{+}^{1,0}|}^{2}\ne 0$ and that it decreases with the magnetic field. Figure 8(a) shows that for low magnetic fields *E*_10_ decreases, while for *B* > 18 T it becomes constant. It explains the reason why in Fig. 9(a) the resonant peak of the absorption coefficient (RPAC) first shows a red shift and then remains stationary at *E*_*P*_ ~ 6 meV. The product ${E}_{10}{|{P}_{+}^{1,0}|}^{2}$ also decreases with the magnetic field and according to Eq. (8), together with the condition ${E}_{10}\gg \Gamma /4$, justifies the decreasing character of the RPAC magnitude in Fig. 9(a). Now let us analyze the results in Fig. 9(b). According to Fig. 8(c), ${|{P}_{-}^{f,0}|}^{2}\ne 0$ (for *f* = 2, …, 14). Note that the curve in Fig. 8(c), which monotonously decreases, is a well-connected combination of different transitions. In Fig. 8(a), the vertical arrows mark the transitions that correspond to the first three intervals of the curve in Fig. 8(c) and show that the transition energy is a growing function with *B*. The behavior of *E*_*f*,0_ (*f* = 2, …, 14) in Fig. 8(a) explains the stable blue shift of the RPAC in Fig. 9(b). The product ${E}_{f0}{|{P}_{-}^{f,0}|}^{2}$ (*f* = 2, …, 14) is dominated by *E*_*f*,0_. In the range of calculated magnetic fields, *E*_*f*,0_ goes from 14.8 meV to 57 meV with an increase of 385% while ${|{P}_{-}^{f,0}|}^{2}$ passes from 75.5 nm^2^ to 35 nm^2^ with a reduction of 46%. In this case ${E}_{f0}{|{P}_{-}^{f,0}|}^{2}$ goes from 1117 meV nm^2^ to 1995 meV nm^2^ with an increase of approximately 178%, which explains that in Fig. 9(b) the magnitude of the RPAC practically doubles when we go from *B* = 0 to *B* = 30 T. Similar analyzes explain the behavior of the OAC in the other panels of Fig. 9. Note that, as stated before, the presence of electric and magnetic fields perpendicular to the axial axis significantly enriches the number of possible transitions manifested in the OAC, as seen in Fig. 9(d–f).

### Off-center donor impurity

Intentional doping with shallow impurities in semiconductor heterostructures, particularly QDs, is a matter that is still in an incipient state of technological development. In general, when doping the system, the impurities are distributed throughout the entire structure with some profiles that obey among other distributions such as the Gaussian, Lorentzian, and in very few opportunities, approximately uniform distributions. In that sense, talking about doping in the center of a CQD is a very strong approximation of the problem. For this reason, in this section, we address the problem of localized impurities throughout the QD volume. As will be seen later, this type of doping gives rise to an impurity band that can be tuned or manipulated as required to modify the optical and electronic properties of a device based on CQD subjected to tilted electric and magnetic fields.

**Figure 10:**
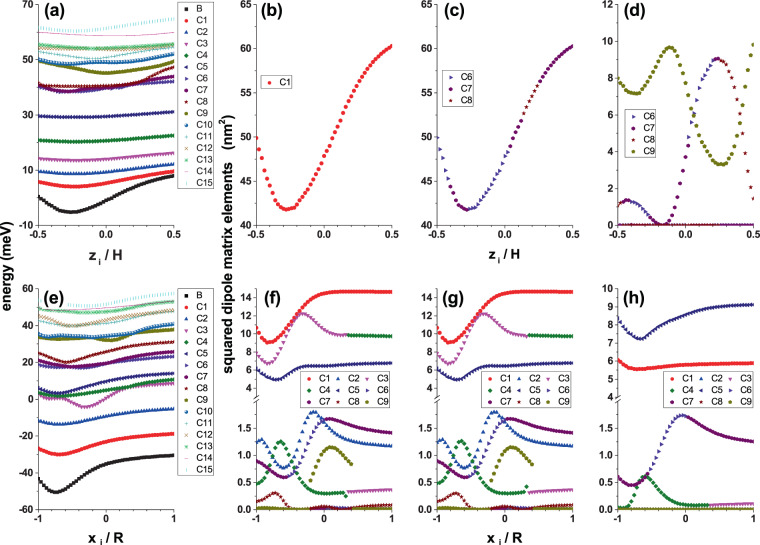
Energy of the off-center impurity lowest confined states (a,e) and Ψ_0_ → Ψ_*n*_ (*n* = 1, 2, 3, …) squared reduced dipole matrix elements (**b**–**d**,**f**–**h**) in a cylindrical GaAs-Al_0.3_ Ga_0.7_ As quantum dot as a function of the axial (**a**–**d**) and radial (**e**–**h**) impurity position with *θ* = *α* = *π*/2 (**a**–**d**) and *θ* = *α* = 0 (**e**–**h**). The results are for *F* = 50 kV/cm, *R* = 20 T, *R* = 20 nm, and *H* = 20 nm. In (**a**,**e**) the label *B* identifies the ground state, whereas the labels *Cn* (*n* = 1, 2, 3, …) are for the excited states. In the squared reduced dipole matrix elements figures, the labels *Cn* (*n* = 1, 2, 3, …) identify the Ψ_0_ → Ψ_*n*_ transitions. The polarizations of the incident photon are as follows: left hand circular polarized radiation (**b**,**f**), right hand circular polarized radiation (**c**,**g**), and *z*-linear polarized radiation (**d**,**h**).

In Fig. 10, we present the energy of the fifteen lowest confined states for a donor impurity in a cylindrical GaAs-Al_0.3_ Ga_0.7_ As QD as a function of the impurity position, considering fixed values of the applied electric and magnetic fields as well as their tilt angles. In Fig. 10(a), the impurity is displaced along the *z*-symmetry axis, while in Fig. 10(e), it is displaced along the *x*-axis. Note that in both cases the impurity displacement direction coincides with that in which the electric and magnetic fields are applied. In Fig. 10(b) and 10(f) are presented the squared dipole matrix elements for left hand circular polarized incident radiation, while the opposite case of circular polarization is reported in Fig. 10(c,g). The Fig. 10(e,h) correspond to the same physical magnitude for *z*-linear polarized incident radiation. In panels 10(a) and 10(e), the energy curves generally have a minimum in the regions −*H*/2 < *z*_*i*_ = 0 and −*R* < *x*_*i*_ = 0, respectively. This effect is visible to at least the three lowest energy states. The excited states of higher energies extend over the entire structure and are difficult to disturb by the electric field influence. The presence of the energy minima, located in the regions detailed above, is because *F* pushes the electronic cloud in the opposite direction to the field, that is, towards the regions −*H*/2 < *z*_*i*_ = 0, in Fig. 10(a), and −*R* < *x*_*i*_ = 0, in Fig. 10(e). As the impurity moves towards these regions, the expected value of the electron-impurity distance decreases, thereby producing an increase in the Coulomb interaction, and consequently giving rise to the minima already described. Referring to the squared reduced dipole matrix elements, reported in the other panels of Fig. 10, the extreme variety of behaviors for such magnitudes is appreciated as different states are considered. While the electric and magnetic fields along the *z*-direction give rise to a single curve of |*P*_+_|^2^ and/or |*P*_−_|^2^ with behaviors that follow the ground state structure (that is, a well-localized minimum), in the case of *x*-oriented fields, to the presence of localized minima in the same region where the ground state becomes minimal, some maximums are added, such as widely visible for transitions 0 → 2 and 0 → 3. This behavior is associated with the fact that the impurity is passing through positions that coincide with nodes and antinodes of the excited state wave function. The product between the ground and excited state wave functions, in those regions where the excited state has nodes and antinodes, also has an oscillating character. When the impurity is located in the regions where the excited state wave function has antinodes, the Coulomb interaction is maximized, while the opposite case occurs when the impurity is located in the region of the excited state wave function nodes. In such regions the product between the two wave functions tends to be suppressed, resulting in the effect of the Coulomb interaction tending to zero. The same argument is useful to explain the oscillating character of |*P*_*z*_|^2^ in Fig. 10(d,h). When comparing Fig. 10(b,c), it is observed that they correspond to two identical curves, which overlap each other. While in Fig. 10(a) the curve comes only from the 0 → 1 transition, the curve in Fig. 10(b) is a combination of 0 → 6, 0 → 7, and 0 → 8 transitions. This later, when we analyze the OAC, will be reflected in results where the resonant structures of the OAC differ by at least an order of magnitude. In Fig. 10(f,g), which have identical squared reduced dipole matrix elements, two sets of well-defined curves can be seen, one of them with values less than 2 nm^2^ and another set in the range 5–15 nm^2^. Since the magnitude of the resonant structure of the OAC is associated with the product between the transition energy and the corresponding squared reduced dipole matrix element, it is clear that in the total coefficient will appear some peaks associated with low energy transition, such as 0 → 1 (given their high values |*P*_+_|^2^ and/or |*P*_−_|^2^), as well as high energy transition peaks (despite their small value of |*P*_+_|^2^ and/or |*P*_−_|^2^). Finally, note that in Fig. 10(f,g) the curve corresponding to the 0 → 9 transition is incomplete. Completing this curve means including in the figure higher-order energy transitions.

**Figure 11:**
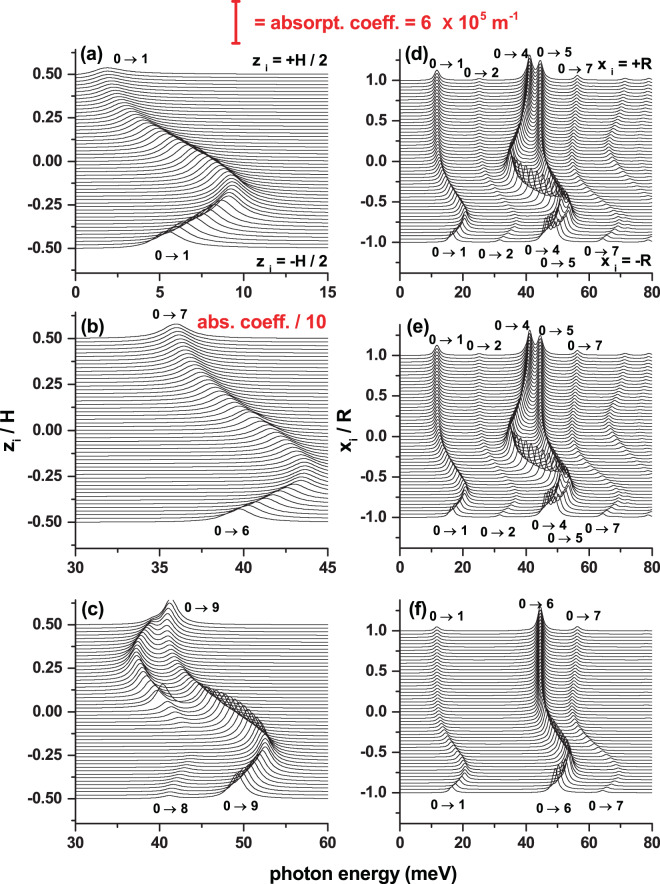
Off-center donor impurity related optical absorption coefficient in a cylindrical GaAs-Al_0.3_ Ga_0.7_ As quantum dot as a function of the incident photon energy and impurity position (axial position-left hand column, radial position-right hand column), with *θ* = *α* = *π*/2 (**a**–**c**) and *θ* = *α* = 0 (**d**–**f**). The results are for *F* = 50 kV/cm, *B* = 20 T, *R* = 20 nm, and *H* = 20 nm. The vertical red bar at the top indicates the magnitude of the optical absorption coefficient. Note that in order to represent all the results in the same scale, in panel (**b**) the OAC has been divided. The polarizations of the incident photon are as follows: left hand circular polarized (**a**,**d**), right hand circular polarized (**b**,**e**), and *z*-linear polarized (**c**,**f**). In all panels, the symbols 0 → *n*, with *n* = 1, 2, 3, … (where 0 corresponds to the initial ground state and *n* is for the final excited one), indicate the allowed optical transitions at *z*_*i*_ = −*H*/2/*x*_*i*_ = −*R* (at the bottom of left/right hand panel) and *z*_*i*_ = +*H*/2/*x*_*i*_ = +*R* (at the top of left/right hand panel).

Taking as input the results presented in Fig. 10, next, in Fig. 11 we present the OAC as a function of the incident photon energy and the impurity position along two directions of the structure: in the left/right hand column panels the results are for the impurity placed along the *z*-/*x*-axis. The CQD is subjected to combined electric and magnetic field effects, which are applied parallel to the axis along which the impurity is displaced. For impurity located along the *z*-axis, note in Fig. 11(a,b) the presence of only one resonant structure, and two well-defined structures in Fig. 11(c), which is in agreement with the number of curves of ${|{M}_{\overrightarrow{\xi }}^{f,0}|}^{2}$ appearing in the upper row of Fig. 10. A similar situation can be seen in Fig. 11(d–f) concerning the second row of Fig. 10. In general, in all the panels of Fig. 11, as the impurity moves from *z*_*i*_ = −*H*/2 to *z*_*i*_ = +*H*/2 (left hand column) or from *x*_*i*_ = −*R* to *x*_*i*_ = +*R* (right hand column), it is observed that the resonant peak initially shows a blue shift followed by a red shift. This effect is clearly explained by the fact that the ground state energy, both in Fig. 10(a,e), is much more sensitive to the impurity position. It can be seen in those that while the ground state curve has a fairly steep minimum (with variations in the order of 20 meV), the curves of the excited states, that have defined minimums, show maximum variations in the order of 15 meV. Comparing the OAC in panels 11(a) and 11(b), where both optical coefficients have identical ${|{M}_{\overrightarrow{\xi }}^{f,0}|}^{2}$ (see Fig. 10(b,c)), it can be seen that for right hand circular polarization the results are of an order of magnitude greater than those corresponding to the left hand circular polarization, which is consistent with the fact that in the first case, such polarization excites transitions to higher-order excited states. After observing the results reported in Figs. 10 and 11, it can be concluded that it is not possible to make predictions about how the optical properties will be for transitions between donor impurity states in CQDs subjected to electric and magnetic fields and under the presence of off-center impurities. In this sense, this research demonstrates by itself its importance to interpret this kind of phenomenology.

**Figure 12:**
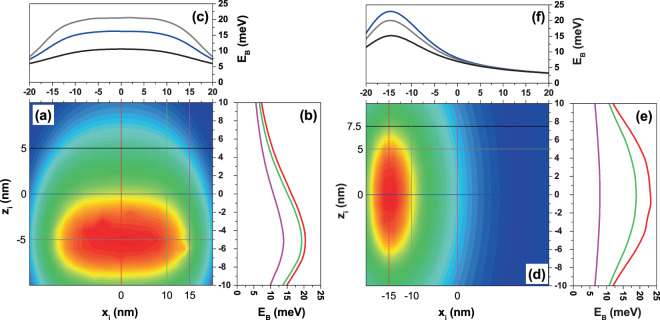
Ground state donor impurity binding energy as a function of the (*x*_*i*_, 0, *z*_*i*_) impurity position in a cylindrical GaAs-Al_0.3_ Ga_0.7_ As quantum dot. The results are for *F* = 50 kV/cm, *B* = 20 T, *R* = 20 nm, and *H* = 20 nm with *θ* = *α* = *π*/2 (**a**–**c**) and *θ* = *α* = 0 (**d**–**f**). Without impurity effects, the ground state energy in (**a**–**c**) is 15.28 meV while in (**d**–**f**) is −27.25 meV.

Given the high multiplicity of variants in our study system (the CQD dimensions (radius and height), the applied fields (electric and/or magnetic fields), the inclination angles of the fields (*θ* and *α*), the absence or presence of the donor impurity, the impurity position, and the circular or linear polarization of the resonant incident radiation), it is extremely difficult to make a full description in a research article of all these possible effects. However, to finish our results and discussion section, we consider that it is important to present, at least, the donor impurities binding energy as a function of the impurity position throughout the *y* = 0 plane and restricting ourselves to the dot region. In Fig. 12(a,b), such results are presented considering fixed electric and magnetic field values applied along the *z*-/*x*-direction. For each binding energy density plot figure as a function of (*x*_*i*_, 0, *z*_*i*_) (Fig. 12(a,d)), three constant *x*_*i*_-values have been chosen (Fig. 12(b,e), respectively) and three constant *z*_*i*_-values also have been depicted (Fig. 12(c,f), respectively). It is evident that Fig. 12(a,c) are symmetric concerning the *x*_*i*_ = 0 line and that Fig. 12(d,e) are symmetric for the *z*_*i*_ = 0 line. The binding energy, in both sets of figures, presents a maximum that is displaced to the QD center in opposite direction to $\overrightarrow{F}$. This binding energy maximum coincides with impurity positions close to the maximum of the ground state probability density of the non correlated electron (without impurity center), that is located towards the flat dot wall at *z* = −*H*/2, in Fig. 12(a), and towards the cylindrical dot wall at *x* = −*R*, in Fig. 12(b). It is important to note that in both figures, depending of the impurity position, the presence of the magnetic field can contributes additively or negatively to the binding energy. In the case of Fig. 12(a), for the impurity in the region |*x*_*i*_| < +*H*/4, the magnetic field reinforces the binding energy while for |*x*_*i*_| > +*H*/4 the magnetic field harms it. A similar analysis is valid for Fig. 12(b), but taking into account the change in the symmetry of the problem.

## Conclusions

Using the effective mass and parabolic band approximations and the numerical finite element method, we have studied the donor impurity related optical and electronic properties in cylindrical GaAs-Al_*x*_ Ga_1−*x*_ As quantum dots under the simultaneous effects of tilted electric and magnetic fields. Considering the several orientations and external field strengths, we report for the first time in the literature: the electronic and shallow donor impurity energy spectra, the ground state binding energy, the impurity related reduced dipole matrix elements for inter-level optical transitions, and the impurity related optical absorption coefficient. We have included a complete analysis of the optical and electronic properties for localized impurities in several positions: (*i*) in the center of the CQD, (*ii*) along the *z*-axis, (*iii*) along the *x*-axis, and (*iv*) at points arbitrarily located on the *y* = 0 plane. From this study, it can be concluded that the presence of tilted electric and magnetic fields and on-center and/or off-center donor impurities are useful tools to enrich the optical and electronic properties of the cylindrical quantum dots due to the break of the cylindrical quantum dot azimuthal symmetry. Our main findings can be summarized as follows: (*i*) in the absence or presence of donor impurity, the ground state energy is always an increasing function of the applied magnetic field, while the excited states show a mixed behavior (in a certain range of the magnetic field they decrease and then start to grow with higher magnetic field strengths), (*ii*) in general, for the high electric field regime a red shift is observed for both the ground and excited states, (*iii*) the ground state binding energy is an increasing/decreasing function of the applied magnetic/electric field, (*iv*) in the presence of an inclined magnetic field with respect to the axial axis of the quantum dot, the number of allowed optical transitions is enriched, as can be deduced from the calculation of the squared reduced dipole matrix elements, and (*v*) the presence of donor impurities located outside the center of the CQD gives rise to extremely intricate energy spectra, squared reduced dipole matrix elements, and optical absorption coefficients that are neither predictable with arguments of symmetry nor phenomenologically, making numerical calculation essential in order to elucidate the physics of these observable. It is important to emphasize that this work constitutes the first report of the optical properties associated with on-center and/or off-center donor impurity states in cylindrical quantum dots with tilted electric and magnetic fields.

## Data Availability

All the files with tables, figures, and codes are available. The corresponding author will provide all the files in case they are requested.
